# Candida vertebral osteomyelitis (CVO) 28 cases from a 10-year retrospective study in France: Erratum

**DOI:** 10.1097/MD.0000000000008910

**Published:** 2017-11-27

In the article, “Candida vertebral osteomyelitis (CVO) 28 cases from a 10-year retrospective study in France”,^[1]^ which appeared in Volume 96, Issue 31 of *Medicine*, Dr. Bruno Fantin's name appeared incorrectly as Fantin Bruno.
